# Nano-carrier DMSN for effective multi-antigen vaccination against SARS-CoV-2

**DOI:** 10.1186/s12951-023-02271-w

**Published:** 2024-01-03

**Authors:** Peng Sun, Bingsheng Cheng, Jiaxi Ru, Xiaoyan Li, Guicun Fang, Yinli Xie, Guangjiang Shi, Jichao Hou, Longwei Zhao, Lipeng Gan, Lina Ma, Chao Liang, Yin Chen, Zhiyong Li

**Affiliations:** 1 School of Basic Medical Sciences, Wenzhou Medical University, Wenzhou, Zhejiang 325000 China; 2 Cixi Biomedical Research Institute, Wenzhou Medical University, Zhejiang, 315302 China; 3 Key Laboratory of Public Health Detection and Etiological Research of Zhejiang Province, Zhejiang Provincial Center for Disease Control and Prevention, Hangzhou, Zhejiang 310051 China; 4 Microscopy Core Facility, Westlake University, Hangzhou, Zhejiang 310030 China

**Keywords:** Antigen nanocarrier, DMSN, Nanoparticles, Vaccine, SARS-CoV-2

## Abstract

The pandemic caused by severe acute respiratory syndrome coronavirus-2 (SARS-CoV-2) has had a profound impact on the global health and economy. While mass vaccination for herd immunity is effective, emerging SARS-CoV-2 variants can evade spike protein-based COVID-19 vaccines. In this study, we develop a new immunization strategy by utilizing a nanocarrier, dendritic mesoporous silica nanoparticle (DMSN), to deliver the receptor-binding domain (RBD) and conserved T-cell epitope peptides (DMSN-P-R), aiming to activate both humoral and cellular immune responses in the host. The synthesized DMSN had good uniformity and dispersion and showed a strong ability to load the RBD and peptide antigens, enhancing their uptake by antigen-presenting cells (APCs) and promoting antigen delivery to lymph nodes. The DMSN-P-R vaccine elicited potent humoral immunity, characterized by highly specific RBD antibodies. Neutralization tests demonstrated significant antibody-mediated neutralizing activity against live SARS-CoV-2. Crucially, the DMSN-P-R vaccine also induced robust T-cell responses that were specifically stimulated by the RBD and conserved T-cell epitope peptides of SARS-CoV-2. The DMSN demonstrated excellent biocompatibility and biosafety in vitro and in vivo, along with degradability. Our study introduces a promising vaccine strategy that utilizes nanocarriers to deliver a range of antigens, effectively enhancing both humoral and cellular immune responses to prevent virus transmission.

**Supplementary Information:**

The online version contains supplementary material available at 10.1186/s12951-023-02271-w.

## Introduction

The COVID-19 pandemic, caused by severe acute respiratory syndrome coronavirus-2 (SARS-CoV-2), has had a profound impact on the global health and economy [1, 2]. SARS-CoV-2 is a member of the family of coronaviruses, and the pandemic resulted in hundreds of millions of infection cases and deaths worldwide [3]. COVID-19 symptoms range from mild to severe, with the most common symptoms including fever, cough, fatigue, headache, body aches, and pneumonia [4, 5]. In severe cases, COVID-19 can progress to acute respiratory distress syndrome (ARDS), organ failure, and even death [6, 7]. The development of an effective SARS-CoV-2 vaccine is crucial for preventing the transmission of and controlling the infection caused by SARS-CoV-2 [8]. The structural spike (S) glycoprotein of SARS-CoV-2 can attach to human angiotensin-converting enzyme 2 (ACE2) for viral entry and infection of targeted host cells [9]. Therefore, the S protein or the receptor‐binding domain (RBD) are the main target antigens for the development of various types of vaccines [8, 10, 11]. The present COVID-19 vaccines, encompassing RNA-based vaccines [12], inactivated vaccines [13], subunit vaccines [14], and various candidates, play a pivotal role in preventing the severity of the disease. However, SARS-CoV-2 variants of concern (VOCs) have undergone significant changes in their transmissibility and virulence, posing challenges and limitations to the use of existing vaccines [15, 16]. Additionally, patients with B-cell/antibody deficiency and a lack of a specific humoral immune response have a significantly higher risk for severe COVID-19 [17, 18]. Therefore, it is necessary to develop a vaccine that is not affected by VOCs and can induce cellular immune responses.

T-cell immunity plays a vital role in controlling SARS-CoV-2 by recognizing and eliminating infected cells [19, 20]. Multiple dominant T-cell epitopes of SARS-CoV-2 have been identified in individuals recovering from COVID-19 [21]. These epitopes have demonstrated high relevance to T-cell immunity against COVID-19 and are implicated in mediating long-term post-infection immunity [21]. However, epitope peptides face challenges in being effectively taken up by host immune cells and in activating immune responses [22, 23]. Effective delivery carriers are crucial for the immune function of epitope peptides [24]. In recent years, nanoparticles have shown promising prospects in the field of carrier technology [25, 26]. DMSN is a type of nanomaterial with a three-dimensional open dendritic framework and center-radial pore structures, exhibiting a higher loading capacity and a more accessible internal surface than those of conventional MSN [27]. Owing to its unique chemical, physical, and structural properties, DMSN has a wide range of potential applications in the field of biomedicine, such as diagnostic bioimaging, drug delivery, cancer treatment, and vaccine development [28, 29]. Numerous studies have demonstrated that DMSN is a promising drug carriers for a wide range of medications, including but not limited to chemotherapeutic agents, immunotherapies, antibiotics, and even drugs with low solubility [30]. In particular, the hierarchical porous structure of DMSN has been employed as a codelivery system, enabling the simultaneous loading of two or more therapeutic agents with varying sizes [27].

Previous studies have indicated that the monomeric RBD of SARS-CoV-2 exhibits limited immunogenicity, leading to the production of low antibody titers [31, 32]. This poor immunogenicity may be due to the small molecular size of the RBD, which can make it difficult for APCs to effectively take it up [31, 33]. Similarly, antigenic epitopes also encounter challenges in activating the host immune system [23]. To enhance the effectiveness of immune responses to the vaccine, we synthesized DMSN as a nanocarrier to codeliver RBD and conserved T-cell epitope peptides of SARS-COV-2, named DMSN-P-R vaccine. The DMSN exhibited a high encapsulation efficiency for antigens of varying molecular sizes and could facilitate the cellular uptake of antigens by APCs, increasing antigen delivery to lymph nodes. The vaccine delivered by the DMSN induced strong humoral immunity with effective neutralizing activity against SARS-CoV-2. Furthermore, the conserved T-cell epitope peptides induced robust IFN-γ T-cell responses. The DMSN had good biocompatibility and biosafety in vitro and in vivo. Therefore, the nanocarrier approach holds great potential for advancing vaccine development against a wide range of infectious diseases.

## Results and discussion

### Synthesis and characterization of the DMSN

**Fig. 1:**
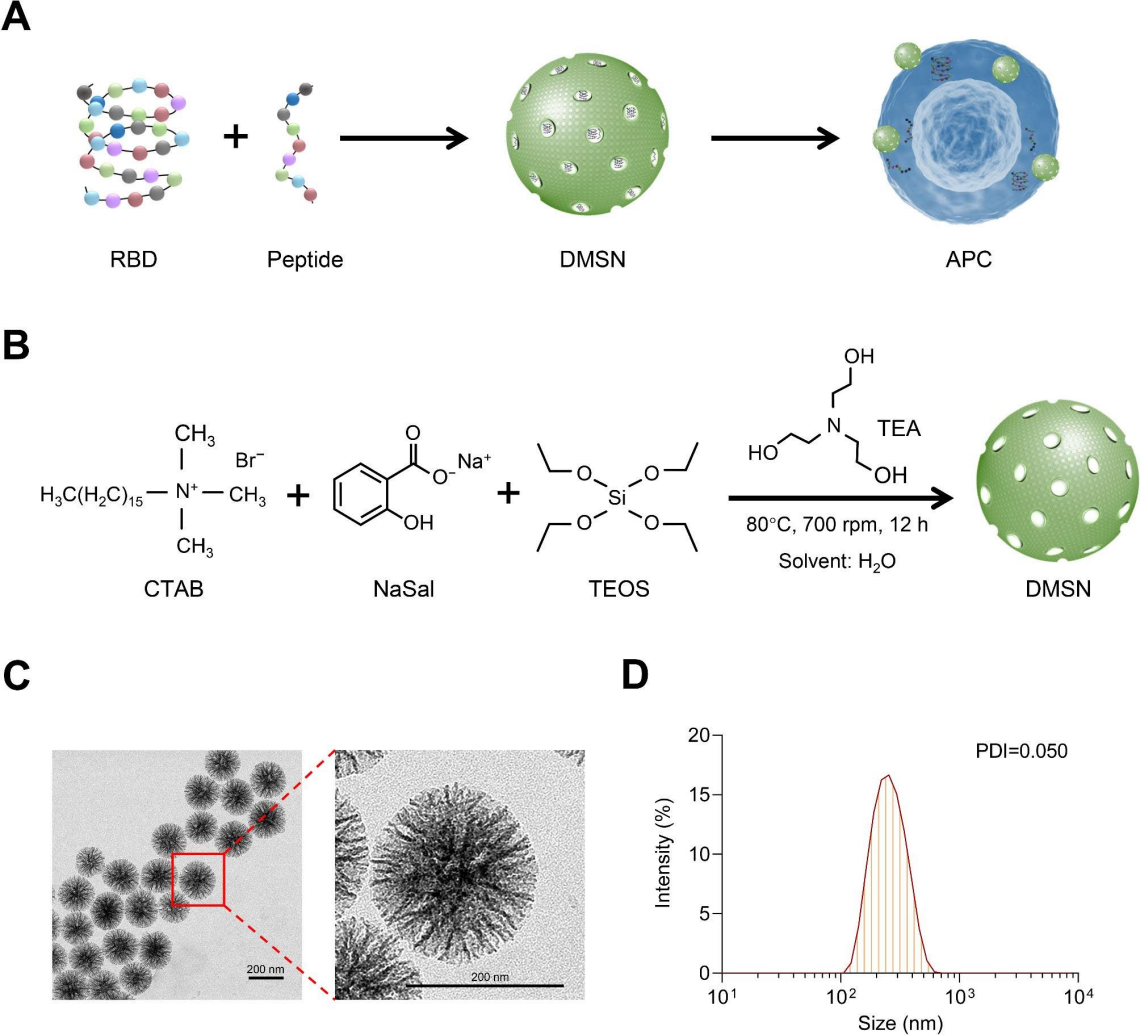
Synthesis and characterization of the DMSN antigen carrier. (**A**) Schematic representation of the study design. The DMSN served as a nanocarrier for delivering both the RBD and conserved T-cell epitope peptides of SARS-CoV-2. (**B**) Synthetic routes of the DMSN. CTAB and NaSal were utilized as the structure-directing agent, with TEOS as the source of silica, and TEA catalyzed the reaction. (**C**) The DMSN structure was examined by TEM. Scale bars, 200 nm. (**D**) Hydrodynamic diameter of the DMSN was determined through DLS measurements

Protein and peptide antigens can be poorly immunogenic because of their small molecular sizes [34, 35]. Porous-structured DMSN has been demonstrated to be effective carriers for various therapeutic agents, including proteins and small-molecule drugs [36]. Owing to their unique structure and properties, DMSN has emerged as highly promising candidate in the field of antigen carriers [30]. We designed DMSN with a hierarchical porous structure as a nanocarrier to enhance the effectiveness of the vaccine by delivering both the RBD and conserved T-cell epitope peptides of SARS-CoV-2 (Fig. 1A). The DMSN with a hierarchical dual-pore structure was synthesized in an aqueous solution using a one-pot sol-gel synthesis approach for vaccine delivery. In this synthesis system, sodium salicylate (NaSal) and cetyltrimethylammonium bromide (CTAB) were employed as costructure-directing agents, triethanolamine (TEA) served as the basic catalyst, and tetraethoxysilane (TEOS) was used as the silica source (Fig. 1B). A variety of synthesis methods have been developed to prepare DMSN, such as oil-water biphase stratification [37], microemulsion templating [38] and a weak template method based on strong counterions [39]. Unlike other synthetic strategies, the approach employed in this study did not involve the addition of organic solvents, which is beneficial for environmental friendliness and large-scale preparation. Using transmission electron microscopy (TEM), we observed that the synthesized DMSN was monodispersed, with a diameter of approximately 200 nm (Fig. 1C). Subsequently, dynamic light scattering (DLS) experiments showed that the synthesized DMSN, with a polydispersity index (PDI) of 0.050, had good uniformity and dispersion (Fig. 1D). The unique characteristics of the DMSN offers significant potential for the loading of large biomolecules.

### Antigen loading onto the DMSN

**Fig. 2:**
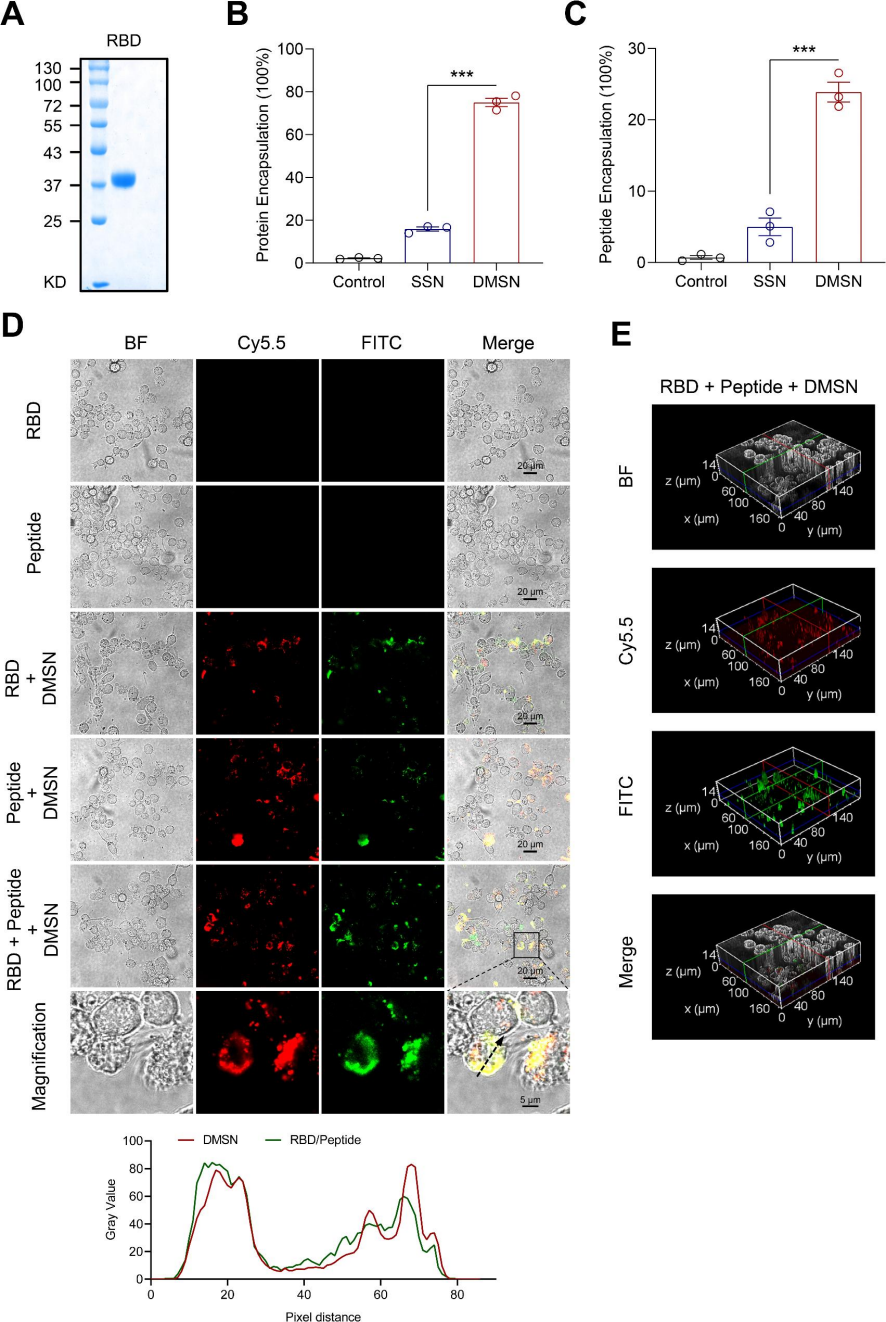
Efficient loading and delivery of antigens by the DMSN. (**A**) The purified RBD of SARS-CoV-2 was analyzed by SDS-PAGE. (**B**–**C**) The DMSN could effectively load the RBD and peptides. The DMSN was mixed with FITC-RBD (**B**) or FITC-peptide (**C**) at a mass ratio of 5:1 and incubated overnight at 4 °C with rotation. After centrifugation, the encapsulation efficiency of the DMSN was calculated according to the protein or peptide content in the supernatant. The data are presented as the mean ± s.e.m. Two-tailed Student’s *t*-test test was used for the statistical analysis. ***p < 0.001. (**D**–**E**) The DMSN facilitated the uptake of the RBD and peptide antigens by THP-1-derived macrophages. FITC-RBD or FITC-peptide was incubated with THP-1-derived macrophages with or without the DMSN. Fluorescence images (**D**) and 3D reconstruction images (**E**) were examined by Leica STELLARIS 5 confocal microscopy. (**D**) The colocalization of the DMSN and RBD/peptide was quantified along a black arrow

To improve its delivery to the immune system, we utilized the DMSN as a carrier for the RBD. We assessed the encapsulation efficiency of the DMSN by using a purified RBD protein (Fig. 2A). The DMSN and RBD were mixed and incubated overnight. The same amount of the RBD mixed with solid silica nanoparticle (SSN), without pores, was used as a control. As shown in Fig. 2B, the DMSN exhibited a higher encapsulation efficiency for the RBD than the SSN, indicating that the DMSN could be an effective candidate for carrying protein antigens. In addition to humoral immunity, T-cell immunity plays a crucial role in controlling SARS-CoV-2 infection, especially in individuals with congenital or acquired B-cell deficiencies who may not produce sufficient levels of antibodies [19, 20]. Therefore, we selected three HLA-DR T-cell epitope peptides derived from the nucleocapsid, envelope, and membrane proteins of SARS-CoV-2 [21], as vaccine targets (Supplementary Fig. 1A). These three peptides are highly conserved and are not affected by VOCs (Supplementary Fig. 1B). Owing to their small molecular weights, peptides often face challenges in being effectively taken up by host immune cells [23, 24]. Therefore, we used the DMSN as a nanocarrier for peptide delivery. As shown in Fig. 2C, the DMSN had a greater peptide encapsulation efficiency than the SSNs. Taken together, the DMSN is an excellent nanocarrier capable of effectively loading proteins and peptides.

### DMSN-mediated antigen delivery

To further determine the delivery capacity of the DMSN, we labeled the DMSN with a fluorescent dye. THP-1-derived macrophages were treated with Cy5.5-labeled DMSN, and subsequently, cellular distribution of the DMSN was examined using fluorescence microscopy. As shown in Supplementary Fig. 2A, a distinct red fluorescence was observed within the cells, indicating that the DMSN was effectively internalized into THP-1-derived macrophages. Next, we assessed the effectiveness of the DMSN in protein delivery. Notably, confocal microscopy analysis revealed that the RBD was barely detectable in THP-1-derived macrophages without the DMSN (Fig. 2D). However, a larger amount of the RBD was observed with the delivery by the DMSN, indicating that the DMSN is an effective delivery carrier for the RBD (Fig. 2D and E). Subsequently, the role of the DMSN in peptide delivery was investigated. As shown in Fig. 2D and E, a significant increase in peptide uptake was observed upon delivery by the DMSN, suggesting that immune cells can effectively take up peptides loaded onto the DMSN. These results collectively indicate that DMSN can effectively facilitate the uptake of proteins and peptides by immune cells.

**Fig. 3:**
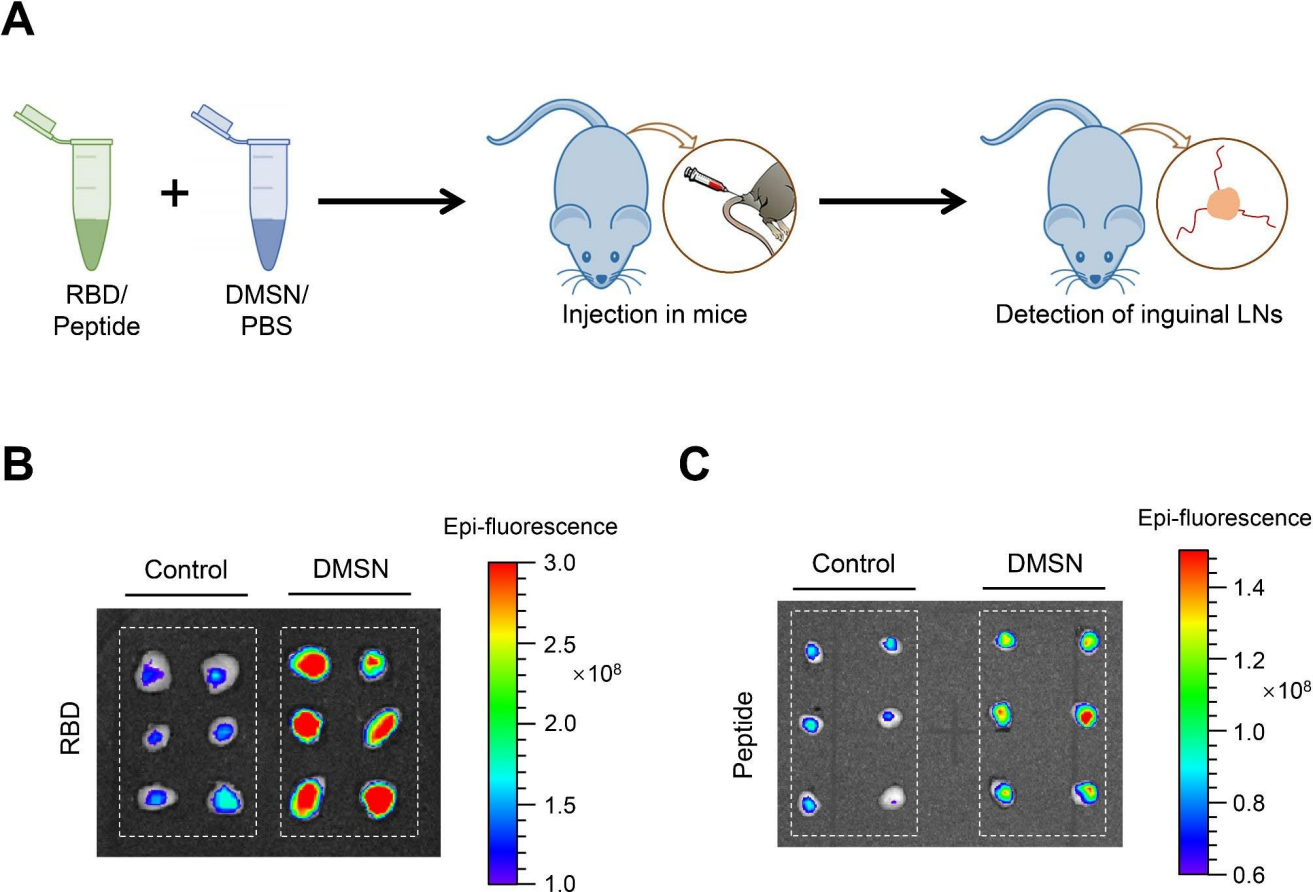
The DMSN facilitates antigen delivery to lymph nodes. (**A**–**C**) Schematic representation of the study design. FITC-RBD or FITC-peptide (10 µg per mouse) with the DMSN (50 µg per mouse) was subcutaneously inoculated into the tail base of BALB/c mice. The same amount of FITC-RBD or FITC-peptide with PBS served as a mock control. At 12 h postincubation, inguinal LNs were isolated and monitored using the IVIS Spectrum

An essential step in adaptive immunity involves APCs, which capture antigens and then migrate to the lymph nodes to engage with T cells [40, 41]. We next examined whether the DMSN could facilitate antigen delivery to lymph nodes using a mouse model. The DMSN was subcutaneously inoculated into the tail base, and the bilateral inguinal lymph nodes (LNs) were isolated (Fig. 3A). Then, the Cy5.5-labeled DMSN in inguinal LNs were analyzed using the in vivo imaging system (IVIS) Spectrum. As shown in Supplementary Fig. 2B-2 C, the DMSN could be presented to bilateral inguinal LNs. Subsequently, a FITC-labeled RBD was incubated with the DMSN, and the mixture was subcutaneously inoculated into the tail base of mice. We found that the incubation with the DMSN significantly increased the RBD delivery to the lymph nodes (Fig. 3B). Consistent with the RBD results, incubation with the DMSN also significantly increased peptide delivery to the lymph nodes (Fig. 3C). Altogether, our data suggest that the DMSN can serve as an effective carrier for protein and peptide delivery.

### Humoral immune responses of the DMSN-based vaccine

**Fig. 4:**
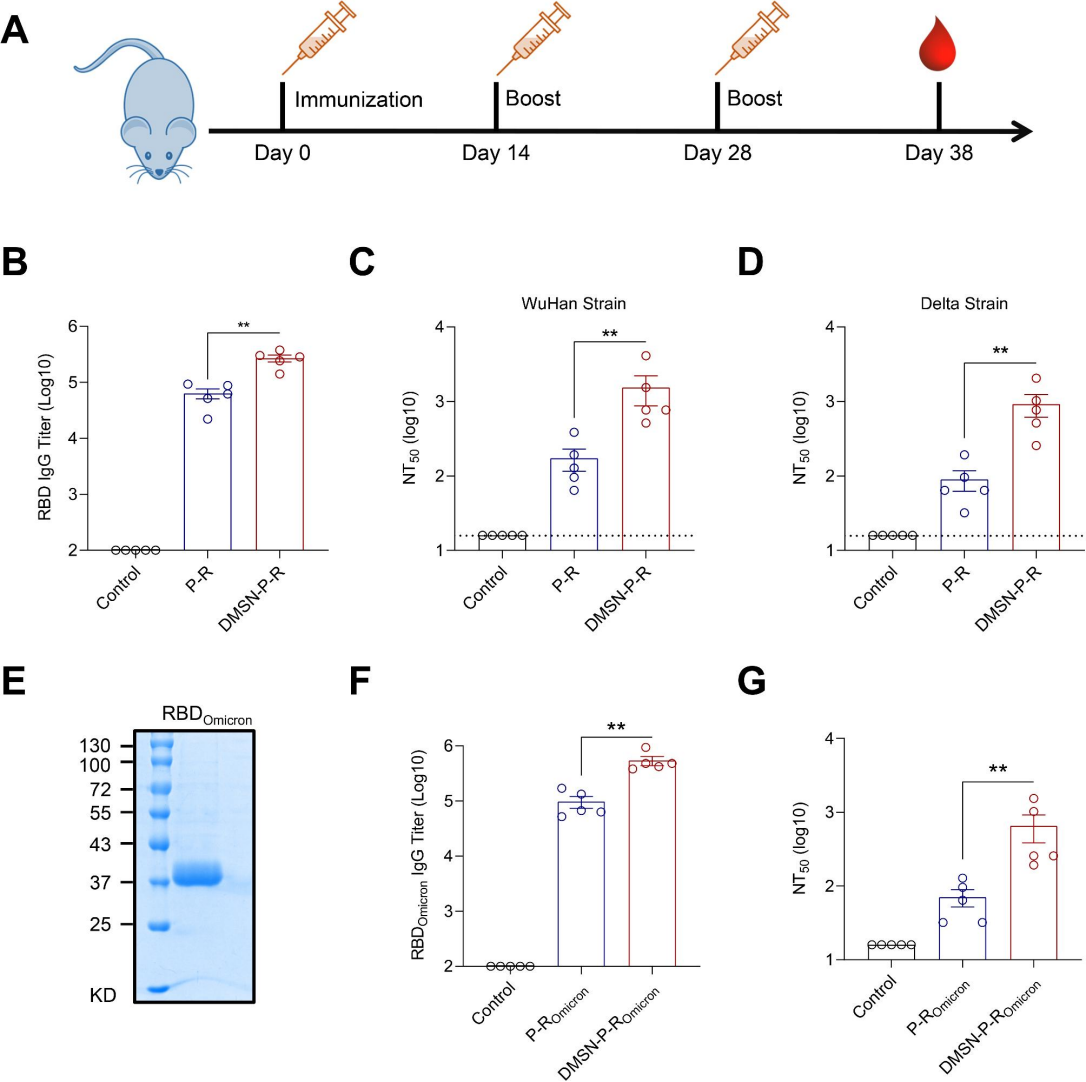
The DMSN-P-R vaccine elicits a strong neutralizing antibody response. (**A**) Schematic of the immunization design. Mice were initially immunized on Day 0, followed by boosters on Day 14 and 28. Sera were collected on Day10 post immunization. (**B**) Sera from immunized mice were analyzed for RBD-specific IgG antibodies using ELISA. (**C**, **D**) Neutralizing antibodies were assessed using live SARS-CoV-2 virus (Wuhan and Delta strain). (**E**) The purified RBD_Omicron_ of SARS-CoV-2 was analyzed by SDS-PAGE. (**F**) Sera were analyzed for RBD_Omicron_-specific IgG antibodies using ELISA. (**G**) Neutralizing antibodies were assessed using live SARS-CoV-2 Omicron BA.1 virus. (**B**–**D**, **F**, **G**) The data are presented as the mean ± s.e.m. A nonparametric Mann-Whitney test was used for the statistical analysis. **p < 0.01

Considering the high antigen-encapsulation efficiency and antigen delivery abilities of the DMSN, we conducted further investigations to determine whether the DMSN could enhance immune responses induced by the RBD and conserved peptides. Alum is the most commonly used adjuvant that improves the effectiveness of vaccines by enhancing immune system response to vaccine antigens, which results in stronger and longer-lasting immunity [42]. Therefore, we loaded the RBD from the Wuhan strain and three HLA-DR T-cell epitope peptides onto the DMSN and then mixed them with an aluminum adjuvant (DMSN-P-R). The same amounts of antigen with the aluminum adjuvant were used as a control (P-R). Next, mice were vaccinated with the DMSN-P-R vaccine using a three-doses immunization schedule (Fig. 4A). Mouse sera were collected on Day 10 after the third immunization, and antibody titers and the potency to neutralize SARS-CoV-2 were analyzed. As shown in Fig. 4B, the group vaccinated with DMSN-P-R showed a significantly higher RBD-specific IgG response than the P-R vaccine group. Subsequently, the neutralizing potency of the sera from the immunized animals was assessed against live SARS-CoV-2 virus. The results showed that the sera from the DMSN-P-R-immunized mice had a higher neutralizing activity against the Wuhan strain than those from the R-P-immunized mice (Fig. 4C), suggesting that the DMSN could enhance the humoral immune response to the vaccine. Next, we assessed the neutralizing potency of the sera against the most popular SARS-CoV-2 variants, including the Delta (B.1.617.2) and Omicron (B.1.1.529) variants. Consistent with the result for the Wuhan strain, DMSN-P-R-vaccinated mice also generated higher titers of neutralizing antibodies (NAbs) against the Delta variant (Fig. 4D). However, the neutralizing ability of the immune sera against the Delta strain was significantly lower than that against the Wuhan strain (Fig. 4C and D), and there was almost no neutralizing ability against the Omicron strain (Supplementary Fig. 3). Consistent with previous reports [43], mutations in the RBD of SARS-CoV-2 variants notably diminish the neutralizing capabilities of antibodies, thereby diminishing the efficacy of existing the RBD-targeting vaccines.

To further validate the role of the DMSN in vaccine immunization, we used the RBD of the SARS-CoV-2 Omicron variant (RBD_Omicron_) with the DMSN to vaccinate mice (Fig. 4E). As shown in Fig. 4F, the group that received DMSN-P-R_Omicron_ vaccination displayed significantly higher titers of the RBD_Omicron_-specific IgG than the group that received P-R_Omicron_ vaccination. Furthermore, the sera of mice immunized with DMSN-P-R_Omicron_ showed a stronger neutralizing effect against the Omicron strain, which was much higher than that in the P-R_Omicron_ group (Fig. 4G). These data indicate that the DMSN could be utilized as a carrier for vaccines to enhance antigen-induced humoral immune responses.

### Cellular immune responses of the DMSN-based vaccine

**Fig. 5:**
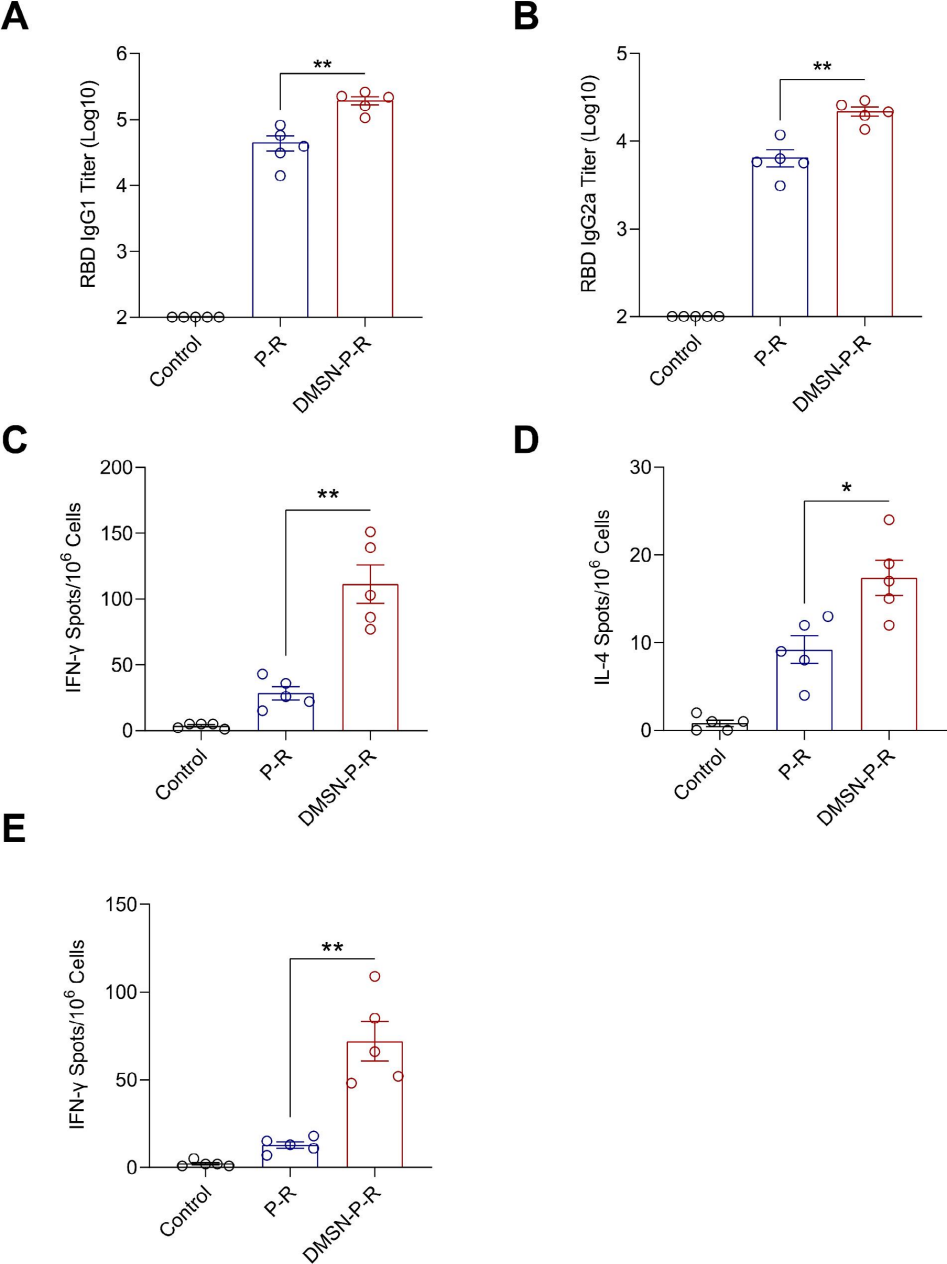
The DMSN-P-R vaccine elicits SARS-CoV-2 specific Th1 and Th2 immune responses. (**A**, **B**) Sera from immunized mice were used to determine the IgG subclasses by ELISA. (**C**, **D**) Mouse splenocytes were isolated on Day 10 post immunization. The ELISPOT assay was performed to measure IFN-γ (**C**) and IL-4 (**D**) secretion from splenocytes following stimulation with the RBD peptides pool. (**E**) The ELISPOT assay was performed to measure IFN-γ secretion from splenocytes following stimulation with conserved T-cell peptides pool. (**A**–**E**) The data are presented as the mean ± s.e.m. A nonparametric Mann-Whitney test was used for the statistical analysis. *p < 0.05, **p < 0.01

We next assessed whether the DMSN could enhance the cellular immune response to the RBD and peptide vaccine. Protein antigens formulated with an alum adjuvant often induce Th2-biased antibody responses rather than strong Th1 responses [44]. IgG1 is generally indicative of Th2 responses, while IgG2a is often associated with Th1 responses [45]. The ELISA results showed that the mice immunized with DMSN-R-P produced higher titers of the RBD-specific IgG1 (Th2) and IgG2a (Th1) than those in the R-P group (Fig. 5A and B). To further validate the subtype of the T-cell immune responses, we performed IFN-γ (Th1) and IL-4 (Th2) enzyme-linked immunospot (ELISPOT) assays [46]. Splenocytes were isolated from the immunized mice and stimulated with the RBD peptide pool. As shown in Fig. 5C and D, the DMSN-P-R group induced stronger IFN-γ and IL-4 responses than the P-R group after stimulation with the RBD peptides, suggesting that the DMSN-carried RBD could produce strong Th1 and Th2 responses. We also investigated the cellular immune responses induced by the three conserved HLA-DR T-cell epitope peptides and found that the DMSN-delivered peptides significantly induced an IFN-γ immune response, indicating a robust peptide-stimulated cellular immune response (Fig. 5E). Given that these three peptides are highly conserved and are not affected by VOCs, the vaccine delivered by that DMSN may exhibit a significant broad-spectrum coverage. Taken together, DMSN-based vaccines are capable of inducing significant cellular immune responses.

### Safety and toxicity

**Fig. 6:**
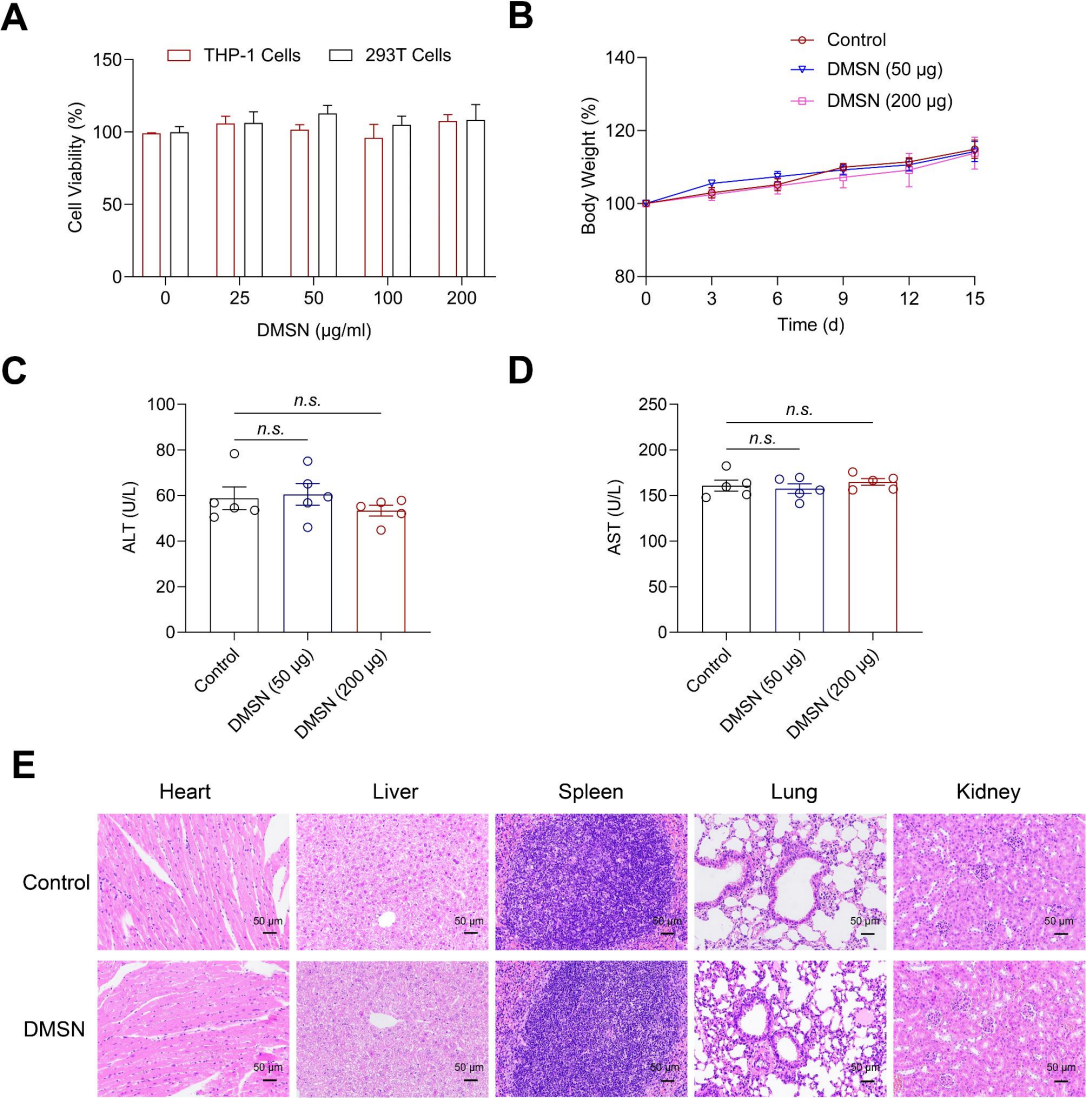
Safety evaluation of DMSN vaccine. (**A**) The viability of THP-1 and 293T cells incubated with the DMSN at various concentrations was assessed using CCK-8. (**B**–**E**) The DMSN was inoculated into BALB/c mice via intramuscular injection. (**B**) The body weight (relative to the initial body weight) of the mice was measured. Fifteen days AfterDMSN inoculation, the serum ALT (**C**) and AST (**D**) were measured. (**E**) H&E staining images of mouse organs were analyzed. Scale bars,50 μm. (**C**, **D**) The data are presented as the mean ± s.e.m. A nonparametric Mann-Whitney test was used for the statistical analysis. *n.s*., not significant (*p ≥ 0.05*)

The possible side effects of administering the DMSN vaccine were subsequently evaluated in vitro and in vivo. As shown in Fig. 6A, the DMSN did not exhibit any notable cellular toxicity in the THP-1 and 293T cell lines, even at a high concentration (200 µg/ml). The body weight of BALB/c mice was monitored after intramuscular injection of DMSN. We found no significant changes in the body weight (Fig. 6B). There was no elevation in the levels of alanine aminotransferase (ALT) or aspartate aminotransferase (AST), indicating that the DMSN does not have any hepatotoxic side effects (Fig. 6C and D). Furthermore, the results of immunohistochemistry (IHC) indicated that the inoculation of the DMSN did not cause any significant pathological abnormalities in the heart, liver, spleen, lungs, or kidney compared to the control group (Fig. 6E). The biodegradability of nanomaterials plays a crucial role in minimizing the potential for long-term accumulation and persistence in the body [47]. The DMSN was incubated in a simulated body fluid (SBF) solution at 37 ℃. After four weeks of incubation, a significant reduction in the DMSN particle size was observed (Supplementary Fig. 4), suggesting that they do not carry a potential risk of accumulation in the body. These data demonstrated that the DMSN exhibited excellent safety and biodegradability. Therefore, the DMSN not only improve the limited immunogenicity of protein and peptide antigens but also have no adverse effects.

## Conclusions

In summary, our research yielded significant advancements in the development of DMSN with exceptional uniformity and dispersion, highlighting its outstanding potential as a vaccine delivery system. These innovative DMSN could efficiently load sizable antigens, including the RBD and peptides of SARS-CoV-2. The immunization results were remarkable, as the vaccine formulations based on the DMSN effectively stimulated a robust humoral immune response and generated higher titers of NAbs against SARS-CoV-2. The RBD and conserved T-cell epitope peptides of SARS-CoV-2 delivered by the DMSN significantly induced a cellular immune response. Furthermore, we conducted a comprehensive evaluation of the safety of the DMSN, which is crucial for their eventual clinical utilization. The assessments revealed excellent DMSN biocompatibility and biosafety in vitro and in vivo. These findings highlight the potential vaccination strategies for nanocarrier with proteins and peptides to induce a host immune response against viral infection.

## Methods

### Mice and cells

Female BALB/c mice aged 6–8 weeks were obtained from Vital River and bred in a specific pathogen-free animal facility at Wenzhou Medical University. The study protocol involving animals was approved by the Institutional Animal Care and Use Committee (IACUC) of Wenzhou Medical University, and all experiments were performed in accordance with their ethical guidelines. The Vero cells and 293T cells were cultured in in Dulbecco’s Modified Eagle’s Medium (DMEM, Gibco) supplemented with 10% heat-inactivated fetal bovine serum (Gibco) and 1% antibiotic-antimycotic (Invitrogen). The THP-1 cells were cultured in Roswell Park Memorial Institute (RPMI) 1640 medium (Gibco) supplemented with 10% heat-inactivated fetal bovine serum. The Vero, 293T and THP-1 cell lines were obtained from the ATCC.

### Synthesis and characterization of DMSN

The DMSN was synthesized according to the following procedures. CTAB (950 mg) and NaSal (300 mg) were added to 60 mL of water and gently stirred in an oil bath at 80 °C until fully dissolved. Then, 150 mg of TEA was added, and the solution was stirred for an additional 30 min. Subsequently, 9 mL of TEOS was added, and the mixture was stirred at 80 °C at a speed of 700 rpm for 12 h. The resulting product was collected by high-speed centrifugation (10,000 rpm, 10 min) and washed multiple times with ethanol to remove any residual reactants. Finally, the obtained product was calcined in air at 600 °C for 8 h, with a heating rate of 3 °C per minute. The synthesized DMSN was characterized using TEM and DLS.

### Protein and peptide encapsulation efficiency

FITC-labeled RBD and peptides were used for the encapsulation experiment. The DMSN was mixed with the antigen at a mass ratio of 5:1 and incubated overnight at 4 °C with rotation. After centrifugation, the protein or peptide content in the supernatant was measured. The encapsulation efficiency was calculated using the following formula: encapsulation efficiency = (initial amount - amount in the supernatant) / initial amount.

### ELISA

The RBD-specific antibody was determined by ELISA assay. SARS-CoV-2 RBD (1 µg/ml) was coated onto plates and incubated overnight at 4 °C. Subsequently, the plates were blocked using a 5% w/v BSA solution for 1 h at room temperature. Serially diluted serum samples from immunized mice were added to the plates and incubated for 2 h. Following washing, IgG-HRP was added to each well, and the plates were further incubated for 1 h. To detect the specific subclasses of IgG, IgG1-HRP or IgG2a-HRP was added. A commercial peroxidase substrate system (SeraCare) was utilized for signal detection, and the optical density (OD) at 450 nm was measured using a microplate reader.

### Mouse immunization

Female BALB/c mice aged 6–8 weeks were immunized via intramuscular injection with a mixture of RBD protein (10 µg/mouse), three conserved T-cell epitope peptides (5 µg/mouse/peptide), DMSN (50 µg/mouse) and aluminum adjuvant (50 µg/mouse). The same amount of RBD, peptide and aluminum adjuvant served as control. The RBD protein and peptide were mixed with DMSN and rotated overnight at 4 °C. Subsequently, the RBD-peptide-loaded DMSN was combined with the aluminum adjuvant. The mice were administered a total of three intramuscular injections at 14-day intervals. Serum samples were collected from the mice to measure the antibody titers.

### ELISpot assay

The ELISpot assay was performed using Mouse IFN-γ and IL-4 ELISpot kits (Mabtech) following the manufacturer’s protocols. Splenocytes obtained from immunized mice were stimulated with either the RBD peptide pool (Sino Biological) or the T-cell epitope peptide pool (Hefei Synth Biotechnology) at a concentration of 5 µg/ml for a duration of 48 h. After stimulation, the cells were removed, and the plates were incubated with biotinylated antibodies for 2 h. Subsequently, the plates were washed five times with PBS (phosphate-buffered saline), followed by the addition of Streptavidin-ALP. To visualize the spots, the substrate BCIP/NBT-plus was added. The spots were enumerated using an ELISpot reader.

### In vivo imaging analysis

BALB/c mice were subcutaneously injected with fluorescent dye-labeled proteins or DMSN at the tail base. The isolated inguinal LNs were imaged using an in vivo imaging system (IVIS) Spectrum (PerkinElmer).

### Biodegradation assay in SBF solution

DMSN (1 µg/ml) were incubated with SBF solutions for four weeks at 37 ℃. Then the degraded-DMSN was detected by TEM.

### Neutralization assay of SARS-CoV-2

A neutralization assay of live SARS-CoV-2 was performed using a cytopathic effect (CPE) assay in a biosafety level 3 laboratory. Briefly, heat inactivated sera samples were serially diluted starting at 1:16. Triplicates of each 50 µl mAb dilution were incubated with the same volume of one hundred 50% Tissue Culture Infectious Dose (TCID_50_) of the SARS-CoV-2 incubated at 37 ℃ for 1 h in 96-well plate. After the incubation, the mixtures were transferred to each well of the plate and then incubated at 37 °C for 3 days. CPEs were confirmed for each well in a blinded fashion by two independent observers. The neutralization titer (NT_50_), defined as the highest sample dilution that protected 50% of the wells, was calculated.

### Statistical analysis

Data are presented as mean ± s.e.m. Two-tailed Student’s *t*-tests were performed for in vitro experiments, while non-parametric Mann-Whitney tests were conducted for animal experiments. Descriptive statistics can be found in the figure legends. All analyses were carried out using the statistical software GraphPad Prism.

### Electronic supplementary material

Below is the link to the electronic supplementary material.

Supplementary Material 1: Additional file 1 of Nano-carrier DMSN for effective multi-antigen vaccination against SARS-CoV-2
