# Supplementary material for: Nano-carrier DMSN for effective multi-antigen vaccination against SARS-CoV-2
Source: J Nanobiotechnology. 2024 Jan 3;22:11. doi: 10.1186/s12951-023-02271-w (PMC10763455; doi:10.1186/s12951-023-02271-w)
Supplement: Supplementary file 1 — Supplementary Material 1: Additional file 1 of Nano-carrier DMSN for effective multi-antigen vaccination against SARS-CoV-2 [file 12951_2023_2271_MOESM1_ESM.pdf]

Supplementary Information

Nano-Carrier DMSN for Effective Multi-Antigen Vaccination Against SARS-CoV-2

A

Peptide ID	Sequence
P1-nuc	GTWLTYTGAIKLDDK
P2-env	FYVYSRVKNLNSSRV
P3-mem	LSYYKLGASQRVAGD

B

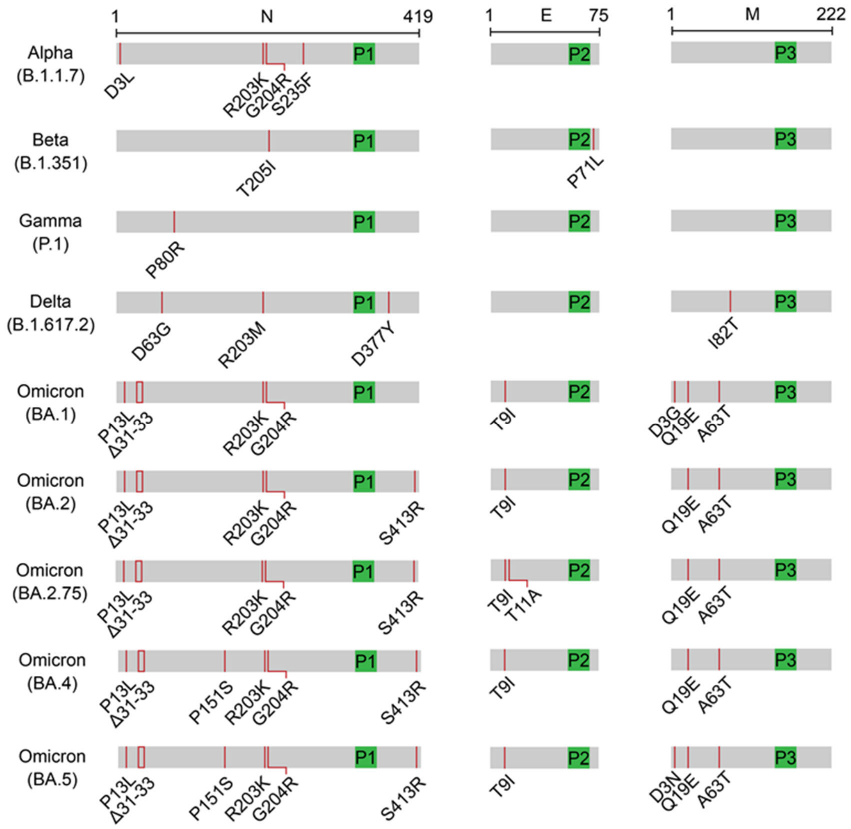

Supplementary Figure 1. Conservation of T-Cell epitope peptides from SARS-CoV-2. (A) The sequences of the three conserved T-cell epitope peptides are derived from the nucleocapsid, envelope, and membrane proteins of SARS-CoV-2. (B) Sequence alignment demonstrated the high conservation of these epitope peptides among different variants of the virus.

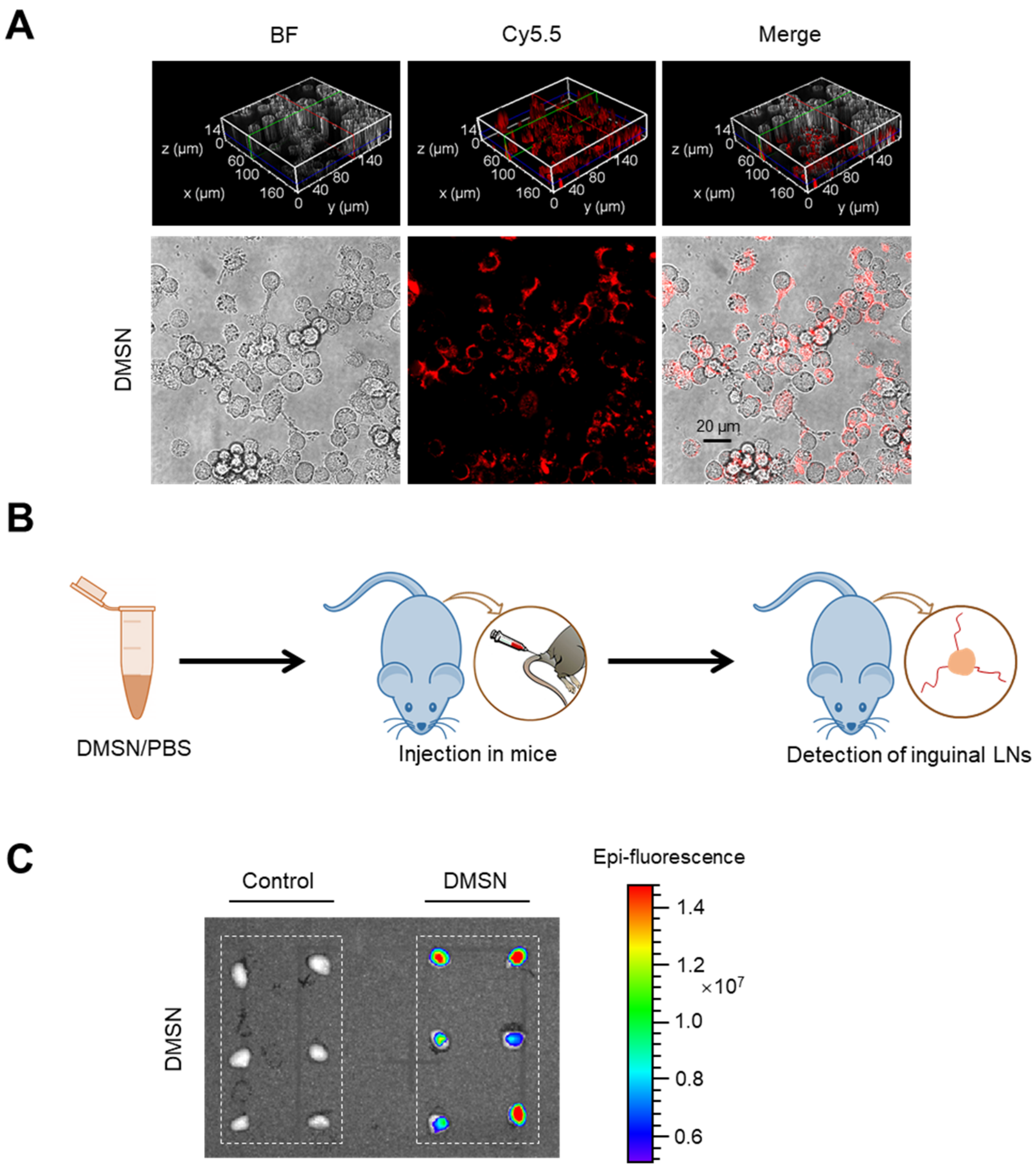

Supplementary Figure 2. Effective internalization of DMSN in THP-1-derived macrophages and presentation to lymph nodes. (A) DMSN was labeled with Cy5.5 fluorescent dye. Cy5.5-DMSN was incubated with THP-1-derived macrophages, and fluorescence images were captured using Leica STELLARIS 5 confocal microscopy. (B-C) Cy5.5-DMSN was subcutaneously injected at the base of the tail in BALB/c mice, with an equivalent amount of PBS serving as mock control. After 12 hours of injection, inguinal lymph nodes were isolated and monitored using an IVIS Spectrum.

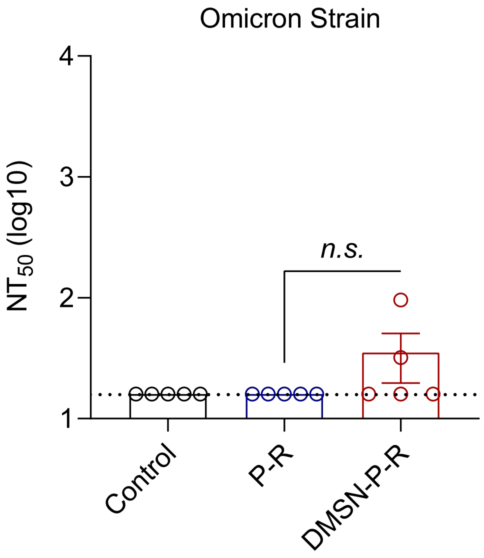

Supplementary Figure 3. Neutralization antibody response against the Omicron BA.1 virus. The neutralizing antibodies in the sera of mice immunized with the DMSN-P-R vaccine were evaluated using live SARS-CoV-2 Omicron BA.1 virus.

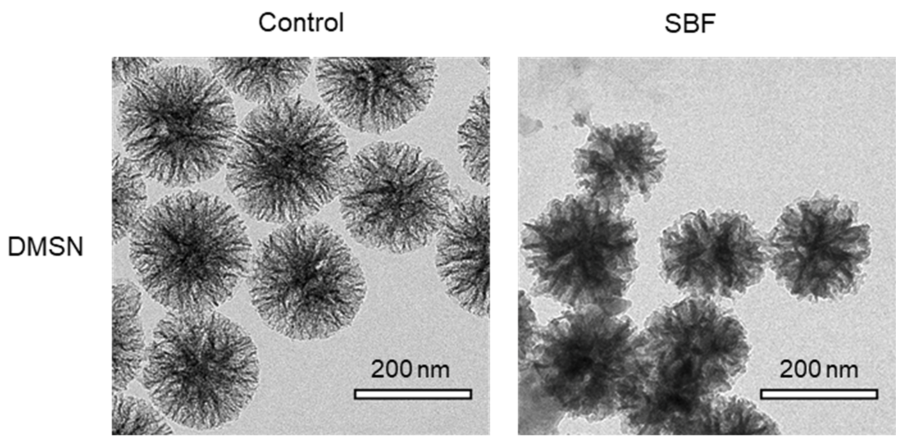

Supplementary Figure 4. DMSN Biodegradability. DMSN was incubated in an SBF solution at 37°C. After four weeks, the DMSN structure was examined using TEM. Scale bars, 200 nm.
